# Behavioral and Psychosocial Interventions for HIV Prevention in Floating Populations in China over the Past Decade: A Systematic Literature Review and Meta-Analysis

**DOI:** 10.1371/journal.pone.0101006

**Published:** 2014-06-25

**Authors:** Xiaona Liu, Vicki Erasmus, Qing Wu, Jan Hendrik Richardus

**Affiliations:** Department of Public Health, Erasmus MC, University Medical Center Rotterdam, Rotterdam, The Netherlands; Alberta Provincial Laboratory for Public Health/University of Alberta, Canada

## Abstract

**Background:**

Floating populations have been repeatedly characterized as “the tipping point” for the HIV epidemic in China. This study aims to systematically summarize and assess the effectiveness of HIV prevention interventions in floating populations in China over the past decade.

**Methods:**

We conducted a systematic search in three international databases for literature published between 2005 and 2012 with condom use as the primary outcome, and knowledge about HIV transmission and prevention and stigma towards HIV-infected individuals as secondary outcomes. The impact of interventions on changing the primary and secondary outcomes was calculated by risk difference (RD). We also performed subgroup analyses and meta-regression based on different study characteristics, using Stata 12.0, for the primary outcome.

**Results:**

Sixteen studies (out of 149) involved 19 different programs and a total of 10,864 participants at entry from 11 provinces in China. The pooled effect estimate of all studies indicated that people participating in HIV-related interventions were 13% more likely to use condoms (95%CI: 0.07, 0.18), however, the effects on increasing condom use exhibited significant heterogeneity across programs (*P*<0.01, *I* ^2^ = 0.93). The meta-regression results suggest that interventions have been significantly less successful in changing condom use in more recent studies (β, 0.14; 95%CI: 0.01, 0.27), adjusted for sexual relationship, study design and follow-up period. Regarding the secondary outcomes, HIV-related interventions were successful at improving knowledge about HIV transmission and prevention (RD, −0.26; 95%CI: −0.36, −0.16 and RD, −0.25; 95%CI: −0.33, −0.16, respectively), and decreasing stigma (RD, 0.18; 95%CI: 0.09, 0.27).

**Conclusions:**

The included studies between 2005 and 2012 indicate that HIV prevention interventions among Chinese floating populations in the past decade were only marginally effective at increasing condom use, but relatively successful at increasing HIV knowledge and decreasing stigma. To avert new infections, novel sexual risk-reduction interventions taking into account the changing socio-economic and cultural situation of Chinese floating populations are urgently needed.

## Introduction

The official estimate of the number of people with HIV/AIDS in China was 780,000 at the end of 2011, and 39,183 new patients were diagnosed with HIV in 2011 [1]. The HIV/AIDS epidemic in China displays unique epidemiological patterns, with a low overall infection rate but high prevalence among certain high-risk populations, and distinctive geographic variations [2]. Historically, injection drug use [3] and commercial blood/plasma collection were the primary sources of HIV infection in China. However, infection through sexual transmission has increased rapidly in recent years. Newly diagnosed HIV cases through sexual transmission increased from 33.1% in 2006 to 76.3% in 2011 [1].

The emergence of large population movement patterns to find better employment opportunities, higher income, and a more attractive lifestyle in cities was driven by large economic disparities since the implementation of the “Open Door” policy in China in 1978 [4], [5]. Individuals from the so-called Chinese floating population are referred to as people who live in a different area than where their household is registered (the “*hukou*” system). The term floating was used not only reflecting a location move between the place of household registration and living, but also embodying the history of moving along the Yangtze River and the Yellow River, two so-called “mother rivers” in China [6]. It was estimated that there were 221 million floating population at the end of 2010, including 160 million rural-to-urban migrants [7]. The Chinese government and international organizations have repeatedly characterized China's floating population as “the tipping point” for the HIV epidemic in China [8], [9]. According to the latest review of 54 studies, approximately 50% of urban patients with HIV have a migratory background. Compared with the HIV prevalence in the Chinese general population, both the floating-out and floating-in populations have a higher risk of HIV infection [5]. These individuals are usually single, low educated, poorly paid, and from less progressive regions of China where sex education remains taboo [10]–[12]. Even when married, people from the floating population are still a recognized risk group because many spend long periods away from their spouse and may purchase, and in some cases sell, sex while away from home [13], [14]. In addition, high mobility of the population has been identified as a major risk factor for facilitating HIV transmission in China, since they frequently shift between jobs and locations, and seasonal visits back home may further spread HIV infection to others [15], [16]. A cross-sectional survey of 1625 Chinese migrant construction laborers, the largest group of the floating populations, revealed that most participants had never used condoms during sex with their stable partners; 14.2% of participants indicated having had sex with a sex worker, but only 35.4% of them reported consistent condom use when doing so [17].

In the absence of an effective and affordable vaccine and due to the non-curative nature of current antiretroviral therapy, behavioral and psychosocial prevention with the goal of limiting risky sexual behaviors remain central to the efforts to decrease the sexual transmission of HIV [18]. Condom use is one of the most efficient means available to reduce the risk of HIV transmission. According to the UNAIDS global report in 2012, the consistent association between behavior change and reduced incidence provides plausible support for the worldwide impact of behavioral change programming in general, although more specific evidence showing which programmatic elements have which effects is urgently needed to help guide wise investment [19]. Moreover, specific guidelines have not been developed for conducting interventions among floating populations in China. Some short-term education programs are launched during holiday periods when many migrants travel back to their home towns, but their impact has not been evaluated [20]. Thus, despite the extent of the HIV epidemic and increasing coverage of behavior change programs, rigorous evaluations of the outcome of any form of behavioral intervention for HIV prevention in floating populations in China are scarce. Finding out whether interventions are effective is critical and challenging.

Various assessments of the effectiveness of HIV/AIDS and/or sexually transmitted infection prevention activities targeted at floating populations in China have been published [21]; most recently, in 2008, Yu Chen *et al.* systematically reviewed the effects on HIV/AIDS interventions [22]. The review included 18 interventions published in Chinese from 1996 up to and including 2006. The studies were not controlled trials, but before-after studies assessing three outcomes of the intervention: 1) knowledge about HIV transmission, 2) knowledge about means of prevention, and 3) general attitudes toward HIV/AIDS patients. The rate difference (RD) was used as an indicator of the effect of the intervention. However, the majority of reviews applied neither a comprehensive evaluation strategy nor clear inclusion criteria, and many of the reviews are out of date. Therefore, a systematic review that incorporates explicit inclusion criteria is needed to assess systematically both behavioral and psychosocial aspects, and to update current knowledge regarding interventions for HIV/AIDS prevention in the Chinese floating populations.

The objectives of this systematic review were to 1) identify and describe HIV prevention interventions in Chinese floating populations over the past decade; 2) summarize and evaluate the effectiveness of interventions to prevent HIV/AIDS in the floating population to increase condom use; and 3) determine the effect of these interventions on knowledge about HIV transmission and prevention and stigma of HIV infection.

## Methods

This review was conducted and reported according to the Preferred Reporting Items for Systematic Review and Meta-Analyses (PRISMA) statement issued in 2010 (Checklist S1) [23]. A review protocol was developed and followed (Protocol S1).

### Data sources and searches

Studies published in Chinese or English between 1 January 2005 and 1 January 2012 were primarily identified electronically by searching the following databases in July 2012: PubMed, China National Knowledge Infrastructure [24], and Wangfang Data. Key words used in the database search were *(“HIV” or “AIDS”) OR (“Sexual behavior” or “Condom use” or “Sexual risk behavior”) AND (“Floating population” or “Migrant worker” or “liúdòng rénkoˇ* *u” in Hanyu Pinyin) AND (“intervention” or “prevention program”) AND (“China” or “Chinese”).* A manual search was performed by replacing terms with related specific words in Chinese, such as “Floating population” with “Restaurant server”, “Miner”, or “Peasant worker”.

### Study selection

The selecting process included a sequence of examining titles, abstracts and full-text. Titles of all articles retrieved from database searches were screened. The abstracts of relevant articles were audited and all studies that could be included were retrieved. We applied the population, intervention, comparison, outcome (PICO) model with respect to criteria for considering studies [25]. Studies were included or excluded at all stages according to the selection criteria on their participants, intervention, study design, and reported outcomes list below. If the same study data were published in both English and Chinese sources, the articles published in Chinese were treated as duplicates and excluded from the review. If the same study was found published at different times, we included the paper published first. Two authors (X.L. & Q.W.) completed this process with agreement over study eligibility.

### Participants

This review focuses on the general floating population in China. The term “floating populations in China” (Chinese: 流动人口; Hanyu Pinyin: liúdòng rénkoˇu) refers to Chinese citizens who live in an area different from the place where their household is registered, in the “*hukou*” system, without any limit on the duration of living outside their registered household place. Conventional high-risk groups that may overlap with the targeted population were excluded, such as men who have sex with men, female sex workers, money boys and drug users, as well as children of migrant workers who may receive school-based HIV prevention programs.

### Interventions

All forms of behavioral and psychosocial interventions designed to promote a decrease in risky sexual behaviors in Chinese floating populations were eligible for inclusion, with no restrictions on the level or mode of delivery. Only interventions conducted in Mainland China with detailed strategies for follow-up were eligible. We subdivided all intervention strategies into four types for comparison: free condom distribution, peer education, general education, or comprehensive campaigns. The first three intervention types are relatively straightforward, applying a single strategy, whereas a comprehensive campaign was defined as an integrated intervention with at least two single strategies.

### Study design

The following study designs were eligible for inclusion: randomized controlled trials (RCT) and controlled before-and-after (CBA) or before-and-after (BA) studies. To reduce the effects of bias due to selective drop-out, we only included studies reporting no significant differences in the socio-demographic characteristics of participants before and after the intervention(s), including age, gender, educational level and marital status. We excluded review papers, non-peer-reviewed local/governmental reports, and conference abstracts and presentations.

### Outcome measures

Four outcomes were identified as the primary or secondary indicators to assess the effectiveness of HIV prevention interventions. Only articles reporting the primary outcome were eligible, secondary outcomes may or may not have been reported in the included studies.

The primary outcome measure was self-reported condom use. Use was defined as a) having used a condom during their last (sometimes referred to as latest) sexual encounter; or b) using a condom usually or every time for sex. When a paper reported both, we chose the first definition as our indicator. Condom use could be reported with regular sex partners and/or casual sex partners. When both were mentioned, we chose the behavior that occurred with the regular sex partner. The original primary outcome was measured as a binary variable on a nominal scale (e.g., Did you use a condom during your last sexual encounter? “Yes” or “No”).

The secondary outcomes were knowledge about HIV transmission and prevention, and stigma towards HIV-infected individuals. Specifically, we examined the level of accurate knowledge that HIV can be contracted by unsafe sex and condom use can decrease the risk of HIV transmission, and the amount of stigma towards HIV-infected individuals, which we broadly defined as an unfavorable attitude or belief directed towards individuals who are living with HIV [26]. The original secondary outcomes were also measured as binary variables (e.g., Can HIV be contracted by unsafe sex? “Yes” or “No”).

### Data collection and analysis

Data from each eligible study, including study period, study site, study design, language, sample size, rate of loss to follow-up, participants' demographic characteristics, intervention strategy, and intervention outcome, were extracted using a pre-designed data extraction form. All data were entered twice before initiating analysis; the accuracy of all data extracted by the main reviewer was checked, including data in tables.

With respect to the quality of the evidence, the Cochrane Collaboration's tool in Review Manger (RevMan, version 5.0) was used to assess bias risk. Selection bias, performance bias, detection bias, attrition bias, and reporting bias were assessed by two reviewers who judged the risk of these biases for each study as ‘low’, ‘high’, ‘unsure’, or ‘unknown’. Consensus was necessary to form a final judgment. We also performed the Begg and Mazumdar rank correlation test to measure potential presence of publication bias in each outcome of studies with Stata software (version 12.0).

Subgroup analyses and multiple-moderator meta-regression were conducted to evaluate the impact of the following factors on the primary outcome: 1) intercourse relationship: behavior within an unstable sexual relationship (commercial sex, casual sex, extramarital sex partner) or within a stable relationship (spouse, long-term partner); one study might present both indicators; 2) study design: RCTs and CBA studies had control groups, whereas BA studies did not have a control group; 3) follow-up period (from starting the intervention to the end of intervention) of less than or more than 6 months; 4) year of publication: before 2010 or during or after 2010; 5) intervention strategy: free condom distribution, peer education, general health education, or comprehensive campaign.

The absolute impact of interventions on the observed risk of primary and secondary outcomes was measured using risk difference (Risk Difference, RD =  *d*_0_/*n*_0_ – *d*_1_/*n*_1_). RD was applied as comparison between effects before and after interventions to designs that lacked a control group, and applied as comparison between intervention and control groups (corrected by baseline effects) to designs with a control group. For studies that provided more than one intervention interval, risk for each interval was assessed separately. Tests of χ^2^ and *I* ^2^ were used to measure heterogeneity, *Z* tests were conducted to measure the effect within groups, and χ^2^ tests were applied to measure the effect across groups. We managed data and synthesized the effect sizes using RevMan 5.0. We conducted subgroup analyses and multiple-moderator meta-regression using Stata 12.0 (weights were the inverse of variance of each RD). Mantel-Haenszel random effects meta-analyses were applied because the intervention effects were assumed to vary across studies.

## Results

**Figure 1:**
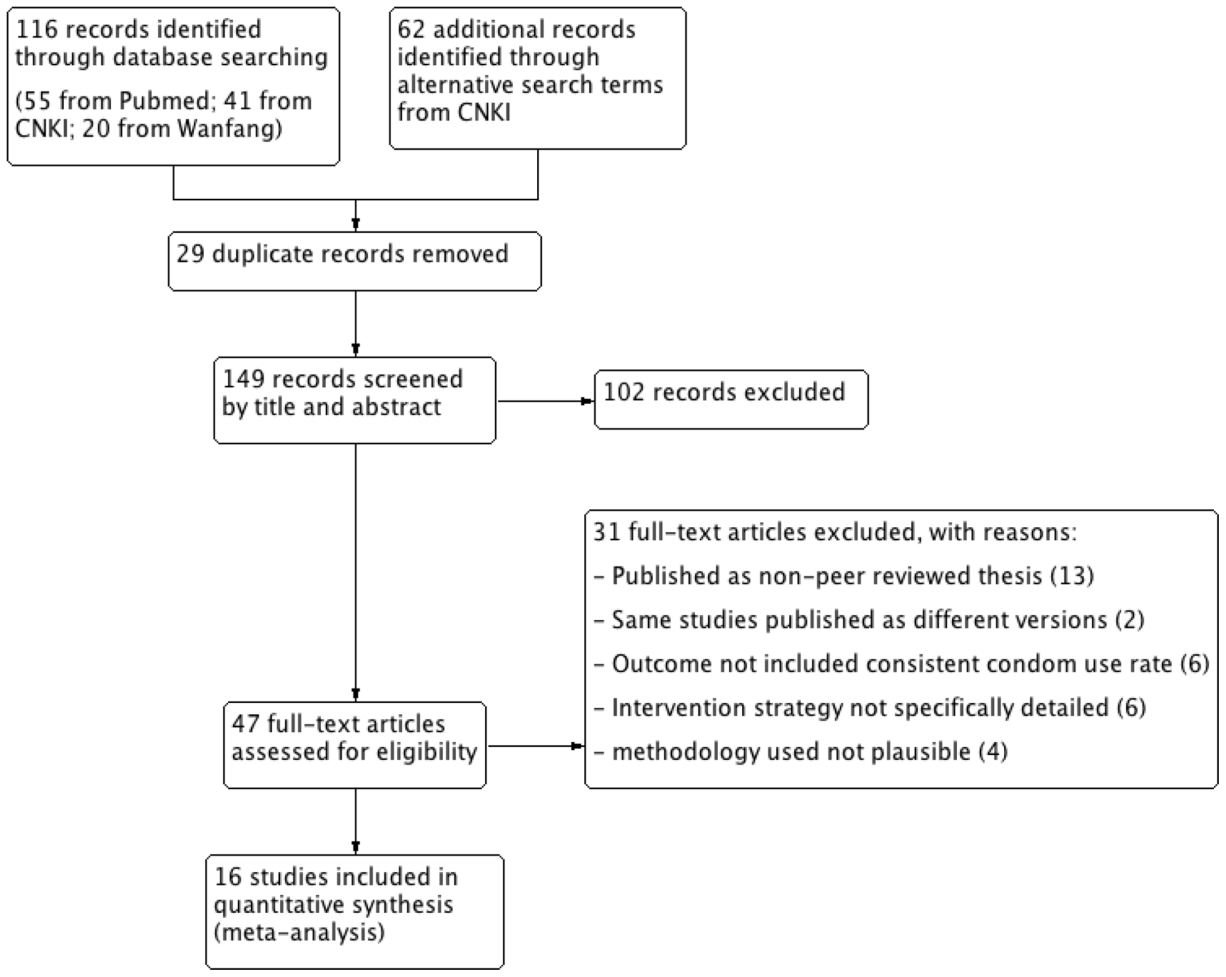
PRISMA flow diagram of the systematic literature reviewing process.

A total of 149 potentially relevant studies were identified in a search of databases (Figure 1). We excluded 102 records at the title or abstract level as they fell outside the scope of this systematic review or did not meet the inclusion criteria, and read the full text of the remaining 47 potentially relevant records. Finally, we included 16 studies published in 13 different journals in our evaluation (Appendix S1) [27]–[42].

### Description of studies

Except one study that was published in English, all included papers were published in Chinese. Four studies, including one RCT and three CBA studies, had a control group. Fifteen studies were BA studies (Table S1). The included studies involved a total of 10,864 participants from 11 different provinces and central municipalities in China. The mean age of participants varied between 18 and 40 years, and the overall educational background was usually lower than secondary school. The majority of workers were male in all samples except three studies carried out in female-only populations [30], [35], [39].

To simplify the comparison of the intervention effects, we recorded every intervention campaign separately. A total of 19 intervention campaigns was recorded in 16 studies, including three studies with two different intervention campaigns [29], [30], [42]. All campaigns were conducted in the decade preceding 2012, although exact starting dates were not given in most studies. Nine of them were published before 2010 and 10 were published in or after 2010. The follow-up period of all campaigns ranged from one month to one year, with a median of six months. Most campaigns supplied general health education as an intervention strategy alone or comprehensively, with formats including general lectures, training of doctors, and the distribution of printed educational materials, including leaflets and posters. One study had free condom distribution as a single strategy [29], and two campaigns conducted peer education as a single strategy [30], [32]. Of the nine comprehensive campaigns, one applied a behavioral theory, namely the protection motivation theory (PMT) [35]. Knowledge, risk perception, self-efficacy, and skills were the main factors included in developing the theory-based intervention, with formats including group discussions, brainstorming, role-playing, homework assignments, games, and lectures. Although four studies indicated having a controlled design, no specific information on the control condition was given.

Regarding outcome measures, five campaigns assessed condom use within casual sex relationships, whereas the remaining 14 campaigns assessed condom use within regular sexual relationships (three assessed them both). Twelve studies reported the level of accurate knowledge about HIV transmission and/or HIV prevention in addition to behavior, and six studies included the level of stigma towards HIV patients.

### Effects of HIV prevention interventions on the primary outcome

**Figure 2:**
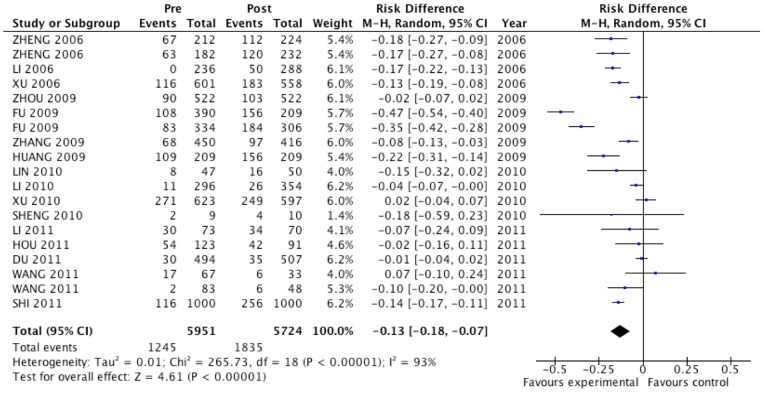
Forest plot of primary effect evaluation for Chinese floating populations: condom use rate.

We applied Mantel-Haenszel random effects meta-analysis to estimate the intervention effect of the studies (Figure 2). The mean rate of condom use prior to intervention for all studies was 0.23±0.11; 10 of 19 campaigns reported no effect on condom use after intervention (*N* = 2337, 39.3%), whereas the pooled effect estimate of all studies suggested that people who participated in a HIV prevention-related intervention were 13% more likely to use condoms (95%CI: 0.07, 0.18). The result of the heterogeneity test showed that the included campaigns varied significantly in their effects on condom use (*P*<0.01, *I* ^2^ = 0.93).

Subgroup analyses were conducted to explore the possible source of the heterogeneity for the primary outcome (Table 1). The impact of interventions on condom use was not significantly different between regular and casual sexual relationships (χ^2^ = 0.19, *P*>0.05). This also applied to controlled and non-controlled studies (χ^2^ = 3.07, *P*>0.05), and to shorter and longer follow-up periods (χ^2^ = 0.47, *P*>0.05). Papers published during or after 2010 reported no significant effect on increasing condom use among the floating population in China (RD, −0.05; 95%CI: −0.1, 0). This is contrary to papers published before 2010, where statistically significant effects were reported (RD, −0.20; 95%CI: −0.29, −0.11; χ^2^ = 8.52, *P*<0.01). Regardless of whether studies were published before or after 2010, the mean rate of condom use at baseline did not differ significantly between studies (0.27±0.07 and 0.28±0.15, respectively; *t* = 0.072, *P*>0.05). Regarding different intervention strategies, although RD results indicated that all four strategies had a positive effect on condom use, their effect demonstrated no significant difference (χ^2^ = 4.64, *P*>0.05); comprehensive campaigns (that were mostly carried out during or after 2010) were relatively less effective (RD, −0.08 compared with RD, −0.18, −0.22 and −0.15). Even controlling for sexual relationships, study design and follow-up period in a weighted multiple-moderator model, publication year remained significantly related to the effect of the interventions on changing condom use (β, 0.14; 95%CI: 0.01, 0.27). The four factors together explained 21.7% of the variance of the effect.

**Table 1:** Subgroup analysis on condom use evaluation for Chinese Floating Population.

Subgroup	No. of campaign		RD ^c^	Heterogeneity	Effect test	Test of RD ^c^
**Relationship**	Stable	14	−0.12 (−0.19, −0.06)	*I* ^2^ = 0.95	*Z* = 3.58^**a^	χ^2^ = 0.19
	Unstable	5	−0.15 (−0.21, −0.08)	*I* ^2^ = 0.64	*Z* = 4.37^**a^	
**Study Design**	RCT & CBA ^b^	4	−0.06 (−0.11, −0.02)	*I* ^2^ = 0.38	*Z* = 4.61^**a^	χ^2^ = 3.07
	BA ^b^	15	−0.14 (−0.20, −0.07)	*I* ^2^ = 0.94	*Z* = 4.08^**a^	
**Published Year**	Before 2010	9	−0.20 (−0.29, −0.11)	*I* ^2^ = 0.94	*Z* = 4.53^**a^	χ^2^ = 8.52^**a^
	In or after 2010	10	−0.05 (−0.10, 0.00)	*I* ^2^ = 0.81	*Z* = 1.98	
**Follow-up**	<6months	6	−0.17 (−0.32, −0.02)	*I* ^2^ = 0.97	*Z* = 2.22^*a^	χ^2^ = 0.47
	≥6months	13	−0.11 (−0.16, −0.07)	*I* ^2^ = 0.84	*Z* = 4.65^**a^	
**Intervention**	Free condoms	1	−0.18 (−0.27, −0.09)	Not applicable	*Z* = 3.98^**a^	χ^2^ = 4.64
	Peer education	2	−0.22 (−0.49, 0.05)	*I* ^2^ = 0.97	*Z* = 1.58	
	General education	7	−0.15 (−0.26, −0.04)	*I* ^2^ = 0.95	*Z* = 2.57^**a^	
	Comprehensive campaign	9	−0.08 (−0.14, −0.02)	*I* ^2^ = 0.86	*Z* = 2.55^*a^	

a. ^*^denotes *P*<0.05; ^**^denotes *P*<0.01.

b. BA: Before-and-after Design; CBA: Controlled Before-and-after; RCT: Randomized Controlled Trail.

c. RD: Risk Difference. Negative RDs imply greater condom use increase for the intervention group than for the comparison group.

### Effects of HIV prevention interventions on secondary outcomes

**Figure 3:**
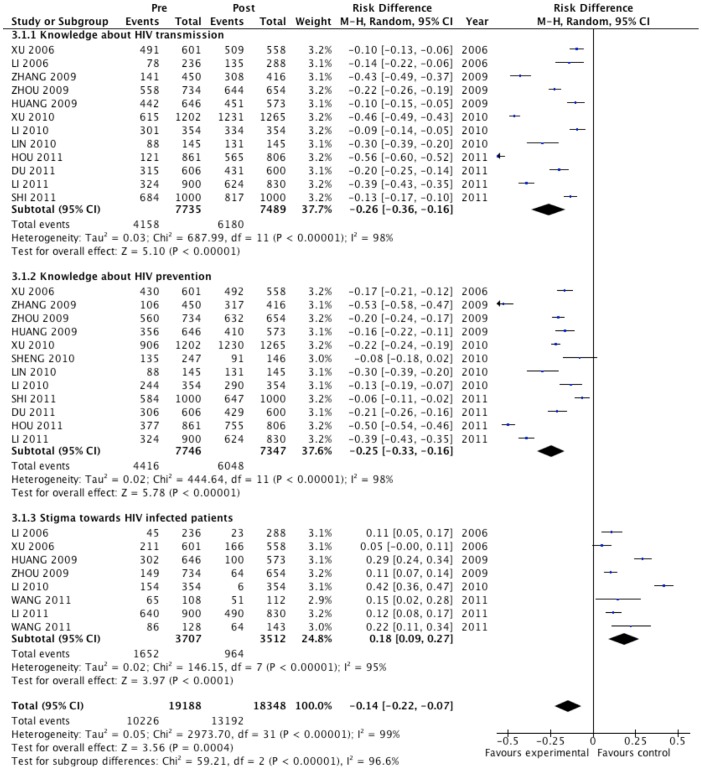
Forest plot of secondary effect evaluation for Chinese floating populations: knowledge and stigma.

Secondary outcomes were reported less frequently (Figure 3). Nonetheless, HIV prevention interventions greatly improved relevant knowledge among the participants, i.e. knowing that HIV can be contracted by unsafe sex and that condom use can decrease the risk of HIV transmission (RD, −0.26; 95%CI: −0.36, −0.16, and RD, −0.25; 95%CI: −0.33, −0.16, respectively), and the effect was significant in all included studies. The estimated pooled effect suggested that people participating in HIV prevention interventions were 18% less likely to exhibit HIV-related stigma (95%CI: 0.09, 0.27).

### Risk of bias

Information on random sequence generation was insufficient in all studies. We identified 75% of the studies as having a high selection bias, whereas 48% of the studies had high attrition bias caused by incomplete outcome data. The risks for performance, detection, and reporting biases varied considerably per study. We also considered other sources of bias, including intervention exposure and delayed exposure, which were lowest in the theory-based study. Results of statistical tests on potential publication bias indicate that the funnel plots were symmetric for all outcomes (*P* = 0.133 in assessing condom use; *P* = 0.485 in assessing knowledge on HIV prevention; *P* = 0.520 in assessing knowledge on HIV transmission and *P* = 0.274 in assessing stigma).

## Discussion

This is the first systematic review to summarize and assess the effectiveness of behavioral and psychosocial HIV prevention interventions among floating populations in China through meta-analysis. The main finding from the included studies between 2005 and 2012 was that HIV prevention interventions among Chinese floating populations in the past decade were only marginally effective at increasing condom use, but relatively successful at increasing HIV knowledge and decreasing stigma.

### Effectiveness of HIV prevention interventions among floating populations in China

This review agrees with some of the findings in the systematic review by Yu Chen *et al.* [22] in 2008, which clarified how HIV prevention interventions increase HIV-related knowledge. However, our review distinguishes the effect on condom use from that of another previous review by Hong *et al.* [2] in 2009. The information published from 2001 to 2008 indicates that HIV-related knowledge significantly increased, as well as condom use, and risky behaviors decreased. The differences with our findings may be explained by the changing of time, variations in definitions of indicators, and in socioeconomic characteristics of participants. The previous review included participants from high-risk populations, such as female sex workers and injection drug users, as well as general population groups, such as students and villagers, in contrast to the specific floating populations studied in this review. In addition, most of the included studies were uncontrolled trials, which are usually considered as effectiveness evidence rather than efficacy evidence. This might also explain the overall modest effect sizes in this study, as effectiveness trials are likely to detect smaller effect sizes than efficacy trials [43].

Subgroup analyses and multiple-moderator analyses for changing condom use indicated that publication year is a possible source of heterogeneity, and the effect on changing condom use was no longer significant in papers published after 2009. However, for the interpretation of this finding we should take into account the correlation between publication year and type of intervention strategy. Single strategies, mostly applied before 2010, were more effective at improving condom use than comprehensive campaigns that were mostly applied in and after 2010. This contradicts the literature on intervention development suggesting that comprehensive interventions should be more effective [44]. The anomaly can be explained by the combination of relatively out of date single strategies now combined in a comprehensive campaign. In other words, more comprehensive campaigns have been conducted in recent years, although they might have generally combined single strategies developed earlier. The current Chinese floating populations have experienced extensive social and economic changes over the last decade. Compared to the older generation of the floating population, the new generation is younger and primarily single when they move to a new city, indicating that they are probably more sexually active and less restricted by their family [45]. In addition, the social norms and rules that previously guided the behavior of people in their villages have been largely abandoned or play a smaller role in a new and more open society [46]. Thus, new intervention strategies should take this changing socio-economic and cultural situation of Chinese floating populations into consideration in order to be effective, as suggested by the literature on intervention development [44]. On the other hand, although the controlled studies (RCT and CBA) didn't show significant differences on intervention outcomes compared to BA studies without the control groups (which may be caused by the limited number of included controlled studies), they generated no significant heterogeneity in this review, indicating that a rigorous study design is important for conducting successful HIV prevention programs among the floating populations in China.

### Implications for future behavioral and psychosocial interventions

A recent WHO systematic review of behavioral interventions targeting adolescents and young adults in developing countries identified 22 school-based studies [47] and 22 community-based behavioral studies [48], [49] that employed experimental or quasi-experimental designs, but none of the studies were conducted in China. The China Ministry of Health recently acknowledged that targeted intervention work for high-risk populations remains stuck at the stage of pilot programs with low coverage [2]. Our systematic review of 16 recent studies indicates the importance of developing HIV/AIDS prevention interventions that take into account the changed socio-economic and cultural situation of the new generation of floating populations. As prior studies have documented, and the results from this review suggest, a change in knowledge alone is not sufficient to reduce high-risk behavior [50] and stigma [51]. Thus, given the relatively high HIV knowledge scores after the conventional promotion strategy in Chinese floating populations (i.e., >50% correct), attention appears to be needed to decrease behavioral risk when developing HIV-related prevention interventions. Most past health education and health promotion campaigns in this review had recognized that their target population has limited health literacy on HIV/AIDS, but they seldom included instruction sessions on how to deal with new obstacles and develop healthy behaviors in the new socio-economic and cultural environment. In order to be effective at promoting behavior that prevents HIV transmission, health education and health promotion campaigns, therefore, should not only provide sufficient information about related healthy behaviors in easily understandable formats and language, but also include sessions on how to deal with various barriers, access to health services, and how to decrease risk in new urban settings [46]. Intervention strategies should also take full advantage of the social network of target populations and create a friendly atmosphere and environment during the implementation of these interventions in order to make them more sustainable. Previous studies have indicated that applying behavioral models at individual level (e.g., the Protection Motivation Theory [35]), and at ecological level (e.g., the Network-Individual-Resource Model [52]), will considerably improve our understanding of the various socio-cognitive and environmental factors of condom use in a structured manner. Further, any successful intervention program must be conducted in close cooperation with cross-cultural and diverse organizations, including CDCs, community health centers, NGOs, and academic institutions.

Increasing attention is given to the important role of the floating population in the HIV epidemic in China, and there are a number of governmental and non-governmental organizations dedicated to the fight against HIV/AIDS among these population groups. To date, besides national and local CDCs, there are two national governmental organizations and seventy-three domestic NGOs working on HIV/AIDS and STD prevention and control in China [53]. In recent years, these organizations have conducted several HIV prevention interventions targeting the floating population across the country, such as the “3 approaches and 3 gifts” [54] “361 model education” [55], and “Caring for migrant workers in construction sites” [56] programs. Strategies in almost all of these interventions consisted of general health education and free condom distribution, as also found in our review. Although we were able gather some descriptive information on these intervention strategies from the websites or in reports of the governmental and non-governmental organizations, we could not identify any clear data on measurement of their effects. Therefore this information could not be included in our study. In two instances there was some reference to improvement of indicators, but without defining the exact outcome effect [55], [57]. Another review also pointed out that there is a lack of reported effect evaluation regarding the achievement of NGOs in preventing HIV transmission in China [58]. To design a successful HIV prevention intervention for the floating population, it is necessary to emphasize the importance of process and effect evaluation.

### Strengths and weaknesses of this review

This study was an update and comprehensive literature review. It not only described the efforts to prevent HIV transmission in Chinese floating population over the past decade, but also applied comprehensive indicators to evaluate their effectiveness. The chosen indicators (condom use, knowledge and stigma) are crucial components of the Knowledge-Attitude-Practice model [59] that was broadly used in developing and evaluating prior health promotion interventions in China. They are also essential factors in the battle against HIV transmission in floating populations, given the marked epidemiological shift in HIV infection from IDU to sexual transmission over the past decade in China. Another strength is that we conducted a meta-analysis to synthesize independent studies and explore differences across studies. The quantified estimate of effectiveness allows policy makers and designers of health education programs to formulate concrete and realistic goals for future behavioral and psychosocial HIV prevention interventions.

However, care must be taken when applying the pooled estimate to general floating populations in China, as the formal definition of this population remains under debate. The floating population of China includes a variety of occupations and the studies in this review are mainly based on one specific occupation, such as construction workers [33]. This systematic review also has some limitations. First, the review is affected directly by the quality of the studies, which not only contained a high selection bias due to the convenient sample approach often applied, but also attrition bias due to participants moving in or out of the area during intervention(s). Second, although socio-demographic characteristics were controlled before and after the intervention(s) in this study, it does not necessarily guarantee controlling for many non-tangible yet profound social and psychological characteristics that may determine the self -selection. Third, the source of heterogeneity on the primary outcome was only explored by subgroup analyses on aspects of study design and interventions. The influences of other factors, especially socio-economic characteristics of participants (such as age, income and educational level), on heterogeneity cannot be elucidated through the data provided in our study. Fourth, we did not explore in depth the factors influencing the secondary outcomes because it was not the main scope of this study. More evidence is needed regarding the contribution of individual intervention elements on their effectiveness at increasing knowledge and decreasing stigma. Finally, we chose to generalize the specific feelings and stigma associated with HIV-infected individuals [26]. However, recent conceptual frameworks have highlighted that important differences exist in the way this stigma may influence the attitudes, behaviors, and experiences of individuals living with HIV compared to individuals without HIV, as well as differences in the relationship with HIV-infected individuals (e.g., family members, colleagues, etc.) [60], [61].

### Future research

We included only one study in this review with a theory-based comprehensive intervention. However, we found potentially high effectiveness for many related programs based on theories about changing health behaviors, such as social cognitive theory. With a better understanding of health behavior theories, the next step should be to apply rigorous study designs with theoretical frameworks to develop effective strategies that meet the needs of the changing socio-economic and cultural situation of the floating population in China.[ref. 19]

## **Data availability**

All data underlying our findings are provided as results in this manuscript and supporting information accompanying this manuscript. The primary data on studied literature are available to all interested researchers on request.

## Supporting Information

Appendix S1 **Study selection form.**
(XLS)

Checklist S1 **PRISMA checklist.**
(DOC)

Protocol S1 **Research protocol of behavioral and psychosocial HIV prevention interventions for floating populations in China.**
(DOC)

Table S1 **Extracted data for description of included studies (** ***n*** **= 16).**
(DOCX)
