# Supplementary material for: Behavioral and Psychosocial Interventions for HIV Prevention in Floating Populations in China over the Past Decade: A Systematic Literature Review and Meta-Analysis
Source: PLoS One. 2014 Jun 25;9(6):e101006. doi: 10.1371/journal.pone.0101006 (PMC4071016; doi:10.1371/journal.pone.0101006)
Supplement: Protocol S1 — Research protocol of behavioral and psychosocial HIV prevention interventions for floating populations in China. (DOC) [file pone.0101006.s003.doc]

**Research Protocol**

Xiaona Liu, M.D., MSC

Vicki Erasmus, PhD

Dr. Jan Hendrik Richardus, M.D, PhD

Infectious Diseases Control Unit, Dept. Public Health, Erasmus MC

**Title:**

Behavioral and psychosocial HIV prevention interventions for floating populations in China: a systematic literature review and meta-analysis

**I. Background**

An HIV/AIDS epidemic has been ongoing in China during the last decades. The current official estimate of people living with HIV was 740,000 (540,000-1000,000) in 2009, while it updated to about 15,982 new patients diagnosed with HIV in 2010. The HIV/AIDS epidemic in China displays unique epidemiological patterns with low overall infection rates but high prevalence among certain high-risk populations with distinctive geographic variations. Historically, intravenous drug use and commercial blood/plasma collection were the primary sources of HIV infection in China; however, infection through sexual transmission is growing the fastest in recent years. Sexually transmitted cases have increased from 7.2% in 2002 to 43.6% of total infections by the end of 2005. Among high-risk populations, the Chinese floating population reached 221 million at the end of 2010, and it mostly consists of migrant workers, with 160 million. According to the head of the National Population and Family Planning Commission, it is estimated that another 300 million people coming from rural areas are expected to migrate for work and live in cities and towns in the next three decades. In China, individuals from these floating populations are referred to as people who live in an area different from the place of their household registration, or "*hukou*" system. They are usually single, poorly paid and from less progressive regions of China, where sex education remains taboo, and where they are immediately exposed to other high-risk groups. Even being married, they are a recognized risk group because many spend long periods away from their spouse, and may purchase, and in some cases sell, sex while away from home. China’s rural-to-urban migrant population has been repeatedly characterized as “the tipping point” for the HIV epidemic in China by the Chinese government and international societies.

While antiretroviral therapy has tremendous life-saving potency, it is expensive and may cause debilitating side-effects for some people. Thus, in the absence of an effective and affordable vaccine and non-curative nature of current antiretroviral therapy, behavioral and psychosocial prevention with the goal of limiting sexual risk behaviors remains central to the efforts to decrease sexual HIV/STI transmissions. However, specific guidelines on conducting interventions among floating populations have not been developed, and no wide-scale interventions exist in China. Some short-term education programs are launched during holiday periods when many migrants travel back to their home towns, but their impact has not been evaluated. Thus, despite the maturity of the HIV epidemic and increasing coverage of interventions, rigorous outcome evaluations of any form of behavioral HIV prevention intervention for floating populations in China are scarce. It is critical and challenging to find out whether they help, harm or are ineffective.

**II. Objectives**

Assessment of the effectiveness of HIV/STI prevention activities targeted at floating populations in China has been published in various publications. Most recently, in 2008, Yu Chen *et al.* systematically reviewed the effects on HIV/AIDS interventions in floating population. The review included 18 interventions, which articles were published in Chinese between 1996 and 2006. The studies were self-control intervention studies and before-after studies, assessing three outcomes of the intervention, including knowledge about HIV transmission, means of prevention, and attitudes towards HIV/AIDS patients. The rate difference (RD) was used as indicator of the effect of the intervention. The majority of reviews neither applied a comprehensive evaluation strategy nor clear inclusion criteria, and many of the reviews are out of date. Therefore, there is a need for a systematic review that incorporates explicit inclusion criteria and that updates the current knowledge base about HIV/AIDS preventive interventions for floating populations in China.

The objectives of the systematic review are to:

1. Identify and describe outcome studies evaluating the effectiveness of HIV prevention interventions on sexual risk for Chinese floating population.
2. Summarize the effectiveness of HIV/AIDS prevention interventions for floating population in increasing consistent condom use, and, if available and possible, HIV knowledge about transmission and means of prevention, and condom use self-efficacy.
3. Identify intervention characteristics associated with effectiveness.
4. Identify gaps in a) subpopulations targeted; b) intervention characteristics incorporated; c) outcomes evaluated; and d) methodological approaches.

**III. Methods and Procedure**

1. **Study design: Systematic literature review and meta-analysis.**

This review will be conducted and reported according to the PRISMA (Preferred Reporting Items for Systematic Review and Meta-Analyses) statement issued in 2010.

1. **Criteria for considering studies for this review**
   1. **Types of studies**

In terms of study design, eligible studies for RCT (randomized controlled trial), CBA (controlled before-and-after) and BA (before-and-after study). Publications written in one of the languages Chinese or English are qualified. To ensure that all research included is relatively new, only publications are included that were published in or after the year 2005.

- 1. **Types of participants**

Apply the population, intervention, comparison, outcome (PICO) model with respect to criteria for considering studies. The term “floating populations in China” ([Chinese](http://en.wikipedia.org/wiki/Chinese_language): 流动人口; [Hanyu Pinyin](http://en.wikipedia.org/wiki/Hanyu_Pinyin): liúdòng rénkǒu), refers to people who live in an area different from the place of their household registration, or "*hukou*" system without floating time limitation. Excluded are children of migrant workers who may receive school-based prevention programs. There are no other limitations on participant characteristics.

- 1. **Type of interventions**

All forms of behavioral and psychosocial interventions designed to promote decrease sexual risk behaviors for Chinese floating populations are eligible for inclusion. No restrictions in level or mode of delivery. For comparisons, no limitations on types of control. No intervention, mild intervention, placebo psychotherapy, routine treatment, or other active STI preventive intervention condition.

- 1. **Types of outcome measures**

Primary outcome: Persistent condom use rate. Considering ways of data collection in Chinese studies, people who self-reported as “having used a condom during the latest sex behavior” or “using a condom usually or every time for sex” will be qualified for this study.

Secondary outcomes: HIV prevention related knowledge and attitude towards HIV/AIDS patients, i.e. 1) correct rate of knowing that HIV can be infected by unsafe sex; 2) correct rate of knowing that condom use can decrease the risk of HIV transmission; and 3) rate of not having stigma towards HIV/AIDS patients.

1. **Search methods for identification of studies**

**3.1 Search strategy**

The primary method of study identification is electronic search, by searching the following databases: *PubMed, China National Knowledge Infrastructure (CNKI) and Wangfang Data* from 2005. Key words used in the database search include *(“HIV” or “AIDS”) OR (“Sexual behavior” or “Condom use” or “Sexual risk behavior”) AND (“Floating population” or “Migrant worker” or “liúdòng rénkǒu” in Hanyu Pinyin) AND ("intervention" or "prevention programme") AND (“China” or “Chinese”).* Manual search will be used as additional other resource.

**3.2 Selection of studies**

Studies are eligible for inclusion in this systematic review if they meet the following criteria: 1) study published in Chinese or English language; 2) HIV prevention intervention was specifically received by floating populations in China; 3) study reported both intervention and effect evaluation; 4) study design such as study site and sample size must be reported; 5) Floating population only refers to people who live in an area different from the place of their household registration, or "*hukou*" system.

Besides, we will exclude review papers, non peer–reviewed local/government reports, and conference abstracts and presentations. If the same study data were published in both English and Chinese sources, the articles published in Chinese language are excluded from the review.

1. **Data collection and analysis procedure**

Data from each eligible study is extracted using a pre-designed data extraction form. The following information will be extracted: first author and published year; study period; study site; study design; language; sample size; follow-up loss rate; participants demographical characteristics; Intervention strategy (including whether the intervention was based on an intervention theory); intervention outcome. All data will be entered twice and before analyses is initiated; the accuracy of all data extracted by the main reviewer will be checked, including data in tables.

Data analyses will be carried out using Review Manger Vision 5 software (RevMan 5. Copenhagen: The Nordic Cochrane Centre. The Cochrane Collaboration, 2008). Mantel-Haenszel random effects meta-analyses will be applied, because it is assumed that the intervention effects will vary across studies.

**IV. Outcome Evaluation**

1. **Assessment of risk of bias in included studies**

With respect to quality of the evidence, we use the Cochrane Collaboration’s tool for assessing risk of bias. Two reviewers discuss and agree about the adequacy of risk of bias for six domains by assigning a judgment of ‘yes’ indicating low risk of bias, ‘no’ indicating high risk of bias, and ‘unclear’ indicating unclear or unknown risk of bias. Criteria set by the Cochrane handbook and adapted to the health promotion field were used to make these judgments.

1. **Measures of intervention effect**

Rate difference (RD) and 95% confidence interval (95% CI) based on the post intervention data will be used as the indicator of intervention effect. Meta-analyses will be taken further to estimate intervention effect separately for primary outcome (condom use) and secondary outcome (two related knowledge points and stigma towards HIV/AIDS patients).

1. **Subgroup analysis and investigation of heterogeneity**

Subgroups will be categorized based on research type: 1) With or without control designed studies; 2) Intervention difference: Common health education, peer education, condom distribution, and theory-based comprehensive intervention; 3) Intervention level: individual-level and community-level; and 4) Follow-up period: short-term (less than 1 month), median-term (more than 1 month and less than half year) and long-term (more than half year).

Heterogeneity tests will be performed using the Cochran Q-test (p<0.10 represents statistically significant heterogeneity) and I^2^ statistic. Factors that are associated with heterogeneities will be investigated in the stratified meta-analysis using meta-regression analysis.

**V. Time table**

| **Study Content** | **Period** | **Study Content** | **Period** |
| --- | --- | --- | --- |
| Needs assessment | 01.10.11 - 20.11.11 | Data analysis | 25.04.12 - 01.06.12 |
| Research proposal | 20.11.11 - 15.12.11 | Paper writing | 01.06.12 - 01.08.12 |
| Systematic literature review | 01.01.12 - 01.03.12 | Paper publish | 01.08.12 - 01.01.13 |
| Data extraction | 01.03.12 - 25.04.12 |  |  |

**VI. References**

1. Hong Y, Li X: **HIV/AIDS behavioral interventions in China: a literature review and recommendation for future research**. *AIDS Behav* 2009, **13**(3):603-613.

2. China MoHo: **2005 Health Statistical Yearbook of China**. In*.* beijing: Ministry of Health, China; 2006.

3. China MoHo: **2010 Health Statistical Yearbook of China**. In*.* beijing: Ministry of Health, China; 2011.

4. 宁艳, 徐鹏, 孙梅, 吕繁: **流动人口艾滋病预防控制的precede模式分析**. *中国卫生政策研究* 2009(08).

5. 刘攀, 汤先忻: **中国流动人口艾滋病流行现状与防控对策**. *MEDICINE AND SOCIETY* 2010(02).

6. 宁艳, 胡俊峰, 吕繁: **流动人口艾滋病健康教育和干预工作现状分析**. *中国健康教育* 2010(04).

7. 李小妹, 周凯娜, 谷利斌: **健康教育对贫困地区农村流动人口艾滋病知识、态度及行为干预效果的研究**. *护理研究* 2010(25).

8. 赵凤霞, 王晓春: **中国流动人口艾滋病危险因素研究进展**. *中国艾滋病性病* 2010(03).

9. He N, Detels R, Chen Z, Jiang Q, Zhu J, Dai Y, Wu M, Zhong X, Fu C, Gui D: **Sexual behavior among employed male rural migrants in Shanghai, China**. *AIDS Educ Prev* 2006, **18**(2):176-186.

10. He N, Wong FY, Huang ZJ, Ding Y, Fu C, Smith BD, Young D, Jiang Q: **HIV risks among two types of male migrants in Shanghai, China: money boys vs. general male migrants**. *AIDS* 2007, **21 Suppl 8**:S73-79.

11. Anderson AF, Qingsi Z, Hua X, Jianfeng B: **China's floating population and the potential for HIV transmission: a social-behavioural perspective**. *AIDS Care* 2003, **15**(2):177-185.

12. China MoHo, UNAIDS, WHO: **2005 update on the HIV/AIDS epidemic and response in China**. In*.*, vol. Jan 24,2006. Beijing: Ministry of Health; 2006.

13. Conant M: **Losing the war on AIDS**. *J Am Acad Dermatol* 2004, **51**(1 Suppl):S47-48.

14. Berg R: **The effectiveness of behavioural and psychosocial HIV/STI prevention interventions for MSM in Europe: A systematic review**. *Euro Surveill* 2009, **14**(48).

15. Rou K, Sullivan SG, Liu P, Wu Z: **Scaling up prevention programmes to reduce the sexual transmission of HIV in China**. *Int J Epidemiol* 2010, **39 Suppl 2**:ii38-46.

16. 虞晨, 孙业桓, 孙良, 王波, 曹红院: **我国流动人口艾滋病预防干预效果的Meta分析**. *中国循证医学杂志* 2008(05).

17. Higgins JP, Green S: **cochrane handbook for systematic reviews of interventions. Version 5.1.0 (updated March 2011).** In*.*: The Cochrane Collaboration; 2011.

18. DL S, SE S, WS R, W R, RB H: **Evidence based medicine: how to practice and teach EBM**. In*.* New York: Churchill Livingston; 2000.

**December 13, 2011**
