# Supplementary material for: Behavioral and Psychosocial Interventions for HIV Prevention in Floating Populations in China over the Past Decade: A Systematic Literature Review and Meta-Analysis
Source: PLoS One. 2014 Jun 25;9(6):e101006. doi: 10.1371/journal.pone.0101006 (PMC4071016; doi:10.1371/journal.pone.0101006)
Supplement: Table S1 — Extracted data for description of included studies ( n = 16). (DOCX) [file pone.0101006.s004.docx]

**Table S1. Extracted data for description of included studies (*n*=16).**

| **Source** | **Study Characteristics** | **Sample Size** | **Demography** | **Intervention Program** | **Outcome** |
| --- | --- | --- | --- | --- | --- |
| **Li,2006**  **[**[**27**](#_ENREF_27)**]** | **Site**: Nanning, Guangxi  **Design**: Before-and-after  **Language**: Chinese | **Before**: 236  **After**: 288 | **Age**(mean): 29.9  **% male**: 84.75  **% >high school**: 2.97  **% married**: 62.71 | **Category**: General health education  **Follow-up period**: 6 months  **Strategies**: Poster publicity; brochures distribution; lectures; video on kill of condom use; health advisory. | Condom use with unstable sexual partner  Knowledge  Stigma |
| **Xu,2006**  **[**[**28**](#_ENREF_28)**]** | **Site**: Yongkang, Zhejiang  **Design**: Before-and-after  **Language**: Chinese | **Before**: 601  **After**: 558 | **Age**(mode): 20-39  **% male**: 53.7  **% >high school**: 8.8  **% married**: 41.1 | **Category**: General health education  **Follow-up period**: 8 months  **Strategies**: Poster publicity: brochures distribution; audio and video propaganda; multiple educational materials dissemination; knowledge quiz. | Condom use  Knowledge  Stigma |
| **Zheng,2006**  **[**[**29**](#_ENREF_29)**]** | **Site**: Xian, Shaanxi  **Design**: Before-and-after  **Language**: Chinese | **Before**: 212  **After**: 224 | **Age**(mean): 28.8  **% male**: 90  **% >high school**: N/A  **% married**: N/A | **Category**: Free condom distribution  **Follow-up period**: 10 months  **Strategies**: poster information about condoms freely available. | Condom use |
|  |  | **Before**: 182  **After**: 232 | **Age**(mean): 28.8  **% male**: 87  **% >high school**: N/A  **% married**: N/A | **Category**: General health education  **Follow-up period**: 10 months  **Strategies**: distribute related educational brochures, and VCDs; lectures on HIV preventive knowledge; advisory or consult by Q-A twice a week. | Condom use |
| **Fu,2009**  **[**[**30**](#_ENREF_30)**]** | **Site**: Guangzhou,  Guangdong  **Design**: Before-and-after  **Language**: Chinese | **Before**: 413  **After**: 381 | **Age**(SD): 26.13±4.98  **% male**: 0  **% >high school**: N/A  **% married**: N/A | **Category**: General health education  **Follow-up period**: 3 months  **Strategies**: Poster publicity; photography shows to promote related knowledge; lectures; pamphlets distribution. | Condom use |
|  |  | **Before**: 356  **After**: 327 | **Age**(SD): 26.78±4.50  **% male**: 0  **% >high school**: N/A  **% married**: N/A | **Category**: Peer education  **Follow-up period**: 3 months  **Strategies**: choose and trained 40 “core” persons as health educator to conduct peer education in their work fields; “core” persons educated others by group discussion and individual communication. | Condom use |
| **Huang,2009**  **[**[**31**](#_ENREF_31)**]** | **Site**: Hefei, Anhui  **Design**: Before-and-after  **Language**: Chinese | **Before**: 646  **After**: 573 | **Age**(mode): 30-40  **% male**: 96.59  **% >high school**: 19.2  **% married**: 86.84 | **Category**: Comprehensive campaign  **Follow-up period**: 1 month  **Strategies**: educational booklets dissemination; poster and bulletin broadcast; related videos and movies watching; peer education; free lecture; free condoms distribution; free advisory and counseling services. | Condom use with sex workers  Knowledge  Stigma |
| **Zhang,2009**  **[**[**32**](#_ENREF_32)**]** | **Site**: Shenzhen  **Design**: Controlled Before-and-after  **Language**: Chinese | **Intervention:**  B: 450 A: 416  **Control**  B: 490; A: 445 | **Age**(mean): 32.73  **% male**: 47.6  **%>high school**: 12.7  **% married**: N/A | **Category**: Peer education  **Follow-up period**: 6 months  **Strategies**: Trained 50 volunteers as peer educators, who educate others about HIV-related knowledge and skills. | Condom use with non-marital sex partner  Knowledge |
| **Zhou,2009**  **[**[**33**](#_ENREF_33)**]** | **Site**: Hefei, Anhui  **Design**: Before-and-after  **Language**: Chinese | **Before**: 943  **After**: 671 | **Age**(mean): 33  **% male**: 93.8  **%>high school**: 17.1  **% married**: 73.4 | **Category**: Comprehensive campaign  **Follow-up period**: 5 months  **Strategies**: free condoms distribution; peer education; mini-media publicity; free consult. | Condom use with stable sexual partner; Knowledge  Stigma |
| **Li,2010**  **[**[**34**](#_ENREF_34)**]** | **Site**: Lantian, Shaanxi  **Design**: Controlled  Before-and-after  **Language**: Chinese | **Intervention**  B: 354; A: 354  **Control**  B: 383; A: 383 | **Age**(mode):18-50  **% male**: N/A  **% ≥high school**: N/A  **% married**: N/A | **Category**: General health education  **Follow-up period**: 4 months  **Strategies**: personal advocacy; pamphlet and booklets distribution; poster; related movies watching; community training of doctor. | Condom use  Knowledge  Stigma |
| **Lin,2010**  **[**[**35**](#_ENREF_35)**]** | **Site**: Beijing  **Design**: Randomize Controlled Trail (RCT)  **Language**: English | **Intervention**  B: 196; A: 145  **Control**  B: 104; A: 81 | **Age**(SD):22.99±9.7  **% male**: 0  **% ≥high school**: 22.0  **% married**: 20.0 | **Category**: Comprehensive campaign  **Theory**: Protection motivation theory (PMT)  **Follow-up period**: 4 months  **Strategie**s: Emphasis on knowledge, negative consequence of high risk behaviors, decision making, goal setting, communication skill, condom negotiation skill, condom use skill, self-efficacy improvement, consensual relationships and empowerment. Format included games, group discussions, videos, role plays, brainstorming and homework assignments. | Condom use  Knowledge |
| **Sheng,2010**  **[**[**36**](#_ENREF_36)**]** | **Site**: Haining, Zhejiang  **Design**: Controlled Before-and-after  **Language**: Chinese | **Intervention**  B: 247; A: 146  **Control**  B: 233; A: 184 | **Age**(mean):39.96  **% male**: N/A  **% >high school**: 1.2  **% married**: 93.5 | **Category**: Comprehensive campaign  **Follow-up period**: 11 months  **Strategies**: conducted for construction workers. Educational materials distribution; focus group discussion; poster and bulletin broadcast; condom distribution and skills counseling; peer education. | Condom use with sex workers  Knowledge  Stigma |
| **Xu, 2010**  **[**[**37**](#_ENREF_37)**]** | **Site**: bars in Beijing  **Design**: Before-and-after  **Language**: Chinese | **Before:** 1202  **After:** 1265 | **Age(mode): 18-35**  **% male: 63.1**  **%≥high school:55.2**  **% married: 20.9** | **Category**: Comprehensive campaign  **Follow-up period**: 4 months  **Strategies**: free condoms distribution; peer education; personal advocacy; lecture and knowledge training; booklets distribution; knowledge quiz with prize. | Condom use  Knowledge |
| **Du, 2011**  **[**[**38**](#_ENREF_38)**]** | **Site**: Chongqing  **Design**: Before-and-after  **Language**: Chinese | **Before**: 600  **After**: 600 | **Age**(Mode):30-40  **% male**:85.31  **%>high school**: 1.82  **% married**: 84.32 | **Category**: Comprehensive campaign  **Follow-up period**: 12 months  **Strategies**: Educational martial distribution; focus group discussion; peer education; free condoms distribution; prized knowledge quiz. | Condom use with spouse  Knowledge |
| **Hou, 2011**  **[**[**39**](#_ENREF_39)**]** | **Site**: Qingdao, Shandong  **Design**: Before-and-after  **Language**: Chinese | **Before**: 861  **After**: 806 | **Age**(SD): 22.0±2.8  **% male**: 0  **%>high school**: 5.9  **% married**: 0 | **Category**: Comprehensive campaign  **Follow-up period**: 6 months  **Strategies**: Reproductive-related education brochures dissemination; Posters; lectures; free condoms distribution; peer education; free counseling hot-line. | Condom use  Knowledge |
| **Li, 2011**  **[**[**40**](#_ENREF_40)**]** | **Site**: Anshang, Liaoning  **Design**: Before-and-after  **Language**: Chinese | **Before**: 900  **After**: 830 | **Age**(SD):34.63±8.67  **% male**: 96.6  **%>high school**: 0.1  **% married**: 24.1 | **Category**: General health education  **Follow-up period**: 12 months  **Strategies**: general community education (poster, educational material distribution in hint) | Condom use with sex workers  Knowledge  Stigma |
| **Shi,2011**  **[**[**41**](#_ENREF_41)**]** | **Site**: Shenyang, Liaoning  **Design**: Before-and-after  **Language**: Chinese | **Before:** 1000  **After:** 1000 | **Age**(SD):34.6±9.3  **% male**: 82.6  **% >high school**: 1.0  **% married**: 65.4 | **Category**: Comprehensive campaign  **Follow-up period**: 6 months  **Strategies**: Educational materials distribution; poster and bulletin broadcast; lectures; health counseling; free condom distribution. | Condom use  Knowledge |
| **Wang,2011**  **[**[**42**](#_ENREF_42)**]** | **Site**: Shenyang, Liaoning  **Design**: Before-and-after  **Language**: Chinese | **Before:** 112  **After:** 112 | **Age**(mode): 30-40  **% male**: 96.4  **%≥high school**: 5.4  **% married**: 82.1 | **Category**: General health education  **Follow-up period**: 6 months  **Strategies**: brochures distribution; posters; video play. | Condom use with spouse  Stigma |
|  |  | **Before:** 143  **After:** 143 | **Age**(mode): 40-50  **% male**: 95.1  **%≥high school**: 5.6  **% married**: 87.4 | **Category**: Comprehensive campaign  **Follow-up period**: 6 months  **Strategies**: brochures distribution; posters; video play; focus group discussion; peer education. | Condom use  Stigma |
